# Relationships between cardiovascular signs and neurological signs in asphyxiated neonates in Ilorin, North Central Nigeria

**DOI:** 10.4314/ahs.v21i2.33

**Published:** 2021-06

**Authors:** Amudalat Issa, Mohammed Baba Abdulkadir, Omotayo Olukemi Adesiyun, Bilkis Owolabi, Habibat Suberu, Kayode Olusegun Alabi, Ruqayat Ronke Bakare

**Affiliations:** 1 Children Specialist hospital, Ilorin, Nigeria; 2 Department of Paediatrics and Child Health, University of Ilorin Teaching Hospital,; 3 Department of Paediatrics and Child Health, University of Ilorin, Nigeria; 4 General hospital, Ilorin, Nigeria

**Keywords:** Cardiovascular signs, Neurological outcomes, Mortality, Perinatal asphyxia

## Abstract

**Background:**

Perinatal asphyxia is a condition associated with multiple organ dysfunctions inclusive of cardiovascular dysfunction. Neurologic predictors of survival have been studied, but little has been reported regarding cardiovascular signs and their role in determining outcome in asphyxia.

**Objective:**

The study aimed to determine the relationship between cardiovascular signs and outcomes in asphyxiated newborns with hypoxic ischaemic encephalopathy.

**Methods:**

This was a cross sectional study involving asphyxiated new-born babies recruited within the first 24 hours of life. Hypoxic ischaemic encephalopathy staging was done using Sarnat and Sarnat staging. All patients had a detailed cardiovascular examination on admission, after initial resuscitation (30 – 60 minutes) into admission, and were followed till final outcome: discharge or death.

**Results:**

Eighty-five asphyxiated new-borns with HIE were studied over seven months. Abnormal cardiovascular-related signs identified in the patients included respiratory distress (64.7%), small volume pulse (57.6%), hypotension (52.9%), hypoxemia (48.2%) and shock (32.9%). Five babies died. None of the clinical signs had a significant relationship with mortality.

**Conclusion:**

Abnormal cardiovascular signs increased with the progression of HIE staging but had no relationship with mortality.

## Introduction

Perinatal asphyxia is defined as progressive hypoxaemia and hypercarbia accompanied by the progressive development of metabolic acidosis.1 It occurs during the perinatal period as a result of compromised placental or pulmonary gas exchange.^1^ It manifests in multiple organs, including the brain, kidneys, the heart and the intestines.^1^ Perinatal asphyxia is associated with insufficient oxygen supply to the cell and redistribution of cardiac output so as to preserve perfusion of the vital organs which include the brain, the heart, and the adrenals.^2^ The peripheral tissues, lungs, intestines, kidneys and other organs are hypo-perfused, in order to favour the vital organs.^3^ However, when the hypoxic ischaemic process becomes severe and prolonged, the vital organs are also affected.^4^ Hypoxic ischaemic encephalopathy (HIE) is the term commonly used to describe the neurological syndrome that occurs following severe perinatal asphyxia.^1^ Hypoxic ischaemic encephalopathy represents the more severe end of the spectrum of organ affection in perinatal asphyxia, with central nervous system (CNS) complications manifesting later on in life as seizure disorders, cerebral palsy and mental retardation.^6^

Perinatal asphyxia is a common neonatal problem that contributes significantly to morbidity and mortality occurring in 1.5/1000 live births in high income countries.^5^ Higher incidences are generally reported in low income countries.^6^ In Nigeria, West and Opara^7^, in a study of 630 neonates admitted over a nine-month period into a Special Care Baby Unit in Port Harcourt reported the incidence of perinatal asphyxia as 24.9%.^7^ A number of studies have been conducted on complications following perinatal asphyxia, especially neurological complications.^8^ Less studied is the cardiac injury as part of the asphyxia syndrome, with a reported incidence of 22.5% to 73.3% in some studies.^9^,^10^ Few studies have described abnormal cardiovascular signs and their relationship with survival in new-borns with hypoxic ischaemic encephalopathy especially in low income countries where they may be the only pointer to myocardial dysfunction. Thus, the present study was conducted to evaluate cardiac dysfunction in perinatal asphyxia using cardiovascular signs. The study also sought to determine the effect of cardiac dysfunction on mortality.

## Material and Methods

This was a cross sectional study in the neonatal unit of the University of Ilorin Teaching Hospital between January and July 2017. The hospital new-born unit serves as a referral centre for patients from neighbouring states. The Neonatal Intensive Care Unit (NICU) monthly admission range between 100 to 150 babies. Perinatal asphyxia among term babies accounts for 10 to 15% of the total admissions. The NICU has facilities for oxygen delivery, ventilatory support (continuous positive airway pressure), non-invasive monitoring, radiant warmers, incubators, phototherapy units and a functioning side laboratory.

A sample size of 85 was calculated using the Kish Leslie formula and prevalence of cardiovascular signs in asphyxiated babies as 73.3%.^10^ Ethical clearance was obtained from the Hospital Ethics Review Committee. Written informed consent was obtained from parents after a detailed explanation of the study.

Inclusion criteria were term AGA neonates with at least stage I HIE, with or without 5th minute Apgar score ≤ 6 admitted within 24 hours of life. The diagnosis of HIE was based on Sarnat and Sarnat staging and the highest stage was assigned.^11^ Babies with congenital heart disease, major congenital anomalies, risk for sepsis, neonatal sepsis, maternal use of medication such as diazepam, opioids within 24 hours of delivery were excluded from the study.

All the babies had their anthropometric parameters measured and were classified based on gestational age using the Lubchenco chart.^12^ Relevant clinical data were obtained. All recruited babies had a detailed cardiovascular system examination recorded at admission after initial resuscitation. The recruitment was done by two of the researchers, who were senior registrars in the department of paediatrics. The blood pressure was measured in the supine position with the neonate quiet or asleep using a non-invasive oscillometric method (WelchAllyn® sphygmomanometer). An appropriate size blood pressure (BP) cuff was used.^13^ The BP cuff was applied to the right upper arm at the level of the right atrium. Two values of the systolic, diastolic and mean arterial blood pressures were read from the monitor and an average recorded. The values were interpreted using the normative oscillometric BP standard for African neonates by Sadoh and Ibhanesebhor.^14^ Values between the 5^th^ and the 95th percentile were recorded as normal, values < 5^th^ percentile as hypotension and above the 95th percentile as hypertension.^13^ Oxygen saturation was measured after initial resuscitation, (about 30 minutes to 1 hour into admission) using a neonatal probe pulse oximeter. Respiratory rate was assessed by counting abdominal and chest movement over one minute with the babies exposed adequately. Other signs of respiratory distress were assessed. The heart rate was counted over 1 minute at the apex with the diaphragm of a stethoscope. Diagnosis of heart failure was made in the presence of tachycardia (heart rate > 160 beats per minute, tachypnoea (respiratory rate > 60 breaths per minute and tender hepatomegaly.^15^ Five of the babies with HIE III in coma, were excluded from assessment of tender hepatomegaly because of difficulties in assessing tenderness in unconscious patients. In addition, diagnosis of shock was made in the presence of poor perfusion (skin colour and capillary refill >3 seconds), small pulse volume with or without hypotension.^16^ The pulse volume was assessed by palpation of the brachial pulse using the pulp of the index and middle fingers. The pulse volume was graded as small or normal.

Data obtained was entered into a computer and analyzed using Statistical Package for Social Sciences (SPSS) software version 20.0 for Windows (SPSS Inc., Chicago, IL, USA). Frequency and mean of categorical and continuous variables were generated respectively. The Chi-square test was used to document relationship between categorical variables. Other statistical tests were used as appropriate. A p value of less than 0.05 was considered statistically significant.

## Results

### General characteristics of the study population

Eighty five asphyxiated neonates (AGA) with hypoxic-ischaemic encephalopathy were recruited for seven months. Twenty-three (27.0%) had HIE stage I, 52 (61.2%) had HIE stage II and 10 (11.8%) had HIE stage III. The median (IQR) age at admission was 1.5 (0.5 to 6.0) hours. The study participants comprised 51 (60.0%) males. The mean ± SD gestational age was 39.5 ± 1.4 9 weeks), birth weight 3.0 ± 0.3 (kg), occipitofrontal circumference 34.7 ± 1.4 (cm), and length 49.0 ± 3.2 (cm).

### Cardiovascular examination findings

The common abnormal cardiovascular findings were respiratory distress in 55 (64.7%), small volume pulse in 49 (57.6%), hypotension in 45 (52.9%), low oxygen saturation (hypoxemia) in 41 (48.2%), reduced peripheral perfusion in 39 (45.9%) and shock in 28 (32.9%). The least common were cardiac murmur [8 (9.4%)], heart failure [5 (5.9%)] and hypertension [1 (1.2%)] as shown in Table 1.

**Table 1:** Cardiovascular examination findings in the subjects

Variable	Frequency (n)	Percentage (%)
**Peripheral perfusion**		
Normal	44	51.8
Reduced	41	48.2
**Central cyanosis**		
Yes	12	14.1
No	73	85.9
**SPO_2_ (%)**		
≤94	41	48.2
95–100	44	51.8
**Respiratory rate (breaths/min)**		
<30	1	1.2
30–60	34	40.0
>60	50	58.8
**Respiratory distress**		
Yes	55	64.7
No	30	35.3
**Pulse volume**		
Normal volume	36	42.4
Small volume	49	57.6
**Heart rate (beats/min)**		
<120	5	5.9
120–160	65	76.5
>160	15	17.6
**Blood pressure**		
Hypotension	45	52.9
Normal	39	45.9
Hypertension	1	1.2
**Shock**		
Yes	28	32.9
No	57	67.1
**Murmur**		
Yes	8	9.4
No	77	90.6
**Tender hepatomegaly**		
Yes	5	6.3
No	75	93.8
**Heart failure**		
Yes	5	6.3
No	75	93.8

### Comparison of cardiovascular signs across disease severity categories

There was an increasing proportion of abnormal cardiovascular signs from HIE I through HIE II and HIE III. Global chi square demonstrated a significant difference in peripheral perfusion, oxygen saturation, pulse volume, pulse rhythm, presence of shock and heart failure, (each p < 0.05), across the three categories (Table 2). Further analysis of the group with HIE I against HIE II and III combined demonstrated peripheral perfusion, pulse volume and shock to be significantly different across the groups, Table 3.

**Table 2:** Comparison of cardiovascular signs across disease severity categories

Variable	HIE I	HIE II	HIE III	Total	*χ* ^2^	*p* value
N=85	n=23 n (%)	n=52 n (%)	n=10 n (%)	N		
**Peripheral perfusion**						
Normal	17 (73.9)^a^	26 (50.0)^a^	1 (10.0)^b^	44	11.569	**0.003**
Reduced	6 (26.1)	26 (50.0)	9 (90.0)	41		
**Central cyanosis**						
Yes	1 (4.3)^a^	7 (13.5)^ab^	4 (40.0)^b^	12	4.695^Y^	0.096
No	22 (95.7)	45 (86.5)	6 (60.0)	73		
SPO_2_ (%)						
<94	8 (34.8)^a^	23 (44.2)^a^	10(100.0)^b^	41	12.733	**0.002**
94–100	15 (65.2)	29 (55.8)	0 (0.0)	44		
**Respiratory rate** **(breaths/min)**						
<30	0 (0.0)^a^	0 (0.0)^a^	1 (10.0)^a^	1	0.374^Y^	0.985
30–60	8 (34.8)	22 (42.3)	4 (40.0)	34		
>60	15 (65.2)	30 (57.7)	5 (50.0)	50		
**Respiratory distress**						
Yes	14 (60.9)^a^	33 (63.5)^a^	8 (80.0)^a^	55	1.208	0.547
No	9 (39.1)	19 (36.5)	2 (20.0)	30		
**Pulse volume**						
Full volume	18 (78.3)^a^	17 (32.7)^b^	1 (10.0)^b^	36	18.421	**<0.001**
Small volume	5 (21.7)	35 (67.3)	9 (90.0)	49		
**Heart rate (beats/minute)**						
<120	0 (0.0)^a^	2 (3.8)^a^	3 (30.0)^a^	5	7.138^Y^	0.129
120–160	18 (78.3)	41 (78.9)	6 (60.0)	65		
>160	5 (21.7)	9 (17.3)	1 (10.0)	15		
**Blood pressure**						
Hypotension	12 (52.2)^a^	27 (51.9)^a^	6 (60.0)^a^	45	1.750^Y^	0.782
Normal	11 (47.8)	25 (48.1)	3 (30.0)	39		
Hypertension	0 (0.0)	0 (0.0)	1 (10.0)	1		
**Shock**						
Yes	1 (4.3)^a^	20 (38.5)^b^	7 (70.0)^b^	28	15.447	**<0.001**
No	22 (95.7)	32 (61.6)	3 (30.0)	57		
**Murmur**						
Yes	1 (4.3)^a^	6 (11.5)^a^	1 (12.5)^a^	8	0.536^Y^	0.765
No	22 (95.7)	46 (88.5)	9 (11.7)	77		
**Heart failure**						
Yes	0 (0.0)^a^	2 (3.8)^ab^	3 (60.0)^b^	5	26.699^Y^	**<0.001**
No	23 (100.0)	50 (96.2)	2 (40.0)	75		

^a,b,c^** Posthoc analysis, parameters with same letter are not different while those with different letters are significantly different.

**Table 3:** Comparison of cardiovascular signs across disease severity categories HIE I against HIE II and III

Variable	HIE I	HIE II & III	Total	*χ* ^2^	*p* value
N=85	n=23 n (%)	n=62 n (%)	N = 85		
**Peripheral perfusion**					
Normal	17 (38.6)	27 (61.3)	44	6.195	**0.013**
Reduced	6 (17.1)	35 (85.4)	41		
**Central cyanosis**					
Yes	1 (8.3)	11(91.7)	12	2.482	0.115
No	22 (30.1)	51 (7.0)	73		
**SPO_2_ (%)**					
<94	8 (19.5)	33 (80.4)	41	2.285	0.131
94–100	15 (34.1)	29 (65.9)	44		
**Respiratory rate (breaths/min)**					
<30	0 (0.0)	1 (100.0)	1	0.805	0.669
30–60	8 (23.5)	26 (76.5)	34		
>60	15 (30.0)	35 (70.0)	50		
**Respiratory distress**					
Yes	14 (25.5)	41 (74.5)	55	0.203	0.652
No	9 (30.0)	21 (70.0)	30		
**Pulse volume**					
Full volume	18 (50.0)	18 (50.0)	36	16.652	**<0.001**
Small volume	5 (10.2)	44 (89.8)	49		
**Heart rate (beats/minute)**					
<120	0 (0.0)	5 (100.0)	5	2.167	0.338
120–160	18 (27.7)	47 (72.3)	65		
>160	5 (33.3)	10 (66.7)	15		
**Blood pressure**					
Normal	11 (28.2)	28 (71.8)	39	0.048	0.827
Abnormal	12 (26.1)	34 (73.9)	46		
**Shock**					
Yes	1(3.6))	27 (96.4)	28	11.671	**0.001**
No	22 (38.6)	35 (61.4)	57		
**Murmur**					
Yes	1 (12.5)	7 (87.5)	8	0.948	0.330
No	22 (28.6)	55 (71.4)	77		
**Heart failure**					
Yes	0 (0.0)	5 (100.0)	5	2.152	0.142
No	23 (29.3)	52 (69.3)	75		

### Comparison of cardiovascular signs across HIE categories

Table 4 shows the comparison of the cardiovascular vital signs across the disease categories. Oxygen saturation was the only parameter with significant relationship across the HIE stages. The mean respiratory rate and heart rate in the participants (all stages) were 66.0 ± 18.94 breaths per minute and 146.06 ± 15.53 beats per minute respectively. Similarly, the systolic, diastolic and mean arterial blood pressure was 58.47 ± 9.70, 28.04 ± 9.03, 38.18 ± 8.18 mmHg respectively.

**Table 4:** Comparison of cardiovascular signs across HIE categories

Variable	HIE I n=23 Mean ± SD	HIE II n=52 Mean ± SD	HIE III n=10 Mean ± SD	*F*	*p*
**Respiratory rate** **(breaths/minute)**	66.9 ± 18.4	66.8 ± 19.2	60.1 ± 19.8	0.544	0.582
**SPO2 (%)**	93.1 ± 7.0	92.5 ± 8.6	80.6 ± 10.5	9.062	**<0.001**
**Heart rate (beats/minute)**	149.2± 13.6	145.6 ± 14.4	141.2 ± 23.8	0.973	0.382
**Systolic BP (mmHg)**	59.3 ± 7.7	58.8 ± 8.7	55.2 ± 16.9	0.662	0.519
**Diastolic BP (mmHg)**	28.6 ± 7.8	28.3 ± 8.4	25.3 ± 14.2	0.519	0.597
**Pulse pressure (mmHg)**	30.7 ± 9.1	30.4 ± 9.4	29.9 ± 9.4	0.260	0.975
**Mean arterial BP** **(mmHg)**	38.8 ± 6.5	38.5 ± 7.3	35.3 ± 14.5	0.728	0.486

### Regression analysis of cardiovascular signs and HIE severity categories

The significant cardiovascular signs in the earlier univariate analyses were subjected to multivariate logistic regression analysis with HIE categories using HIE I as reference category against HIE II and III combined. Pulse volume was the only significant cardiovascular parameter with an independent relationship (four times likelihood) among subjects with HIE II and III compared with HIE I. (Table 5)

**Table 5:** Regression analysis of HIE severity categories with selected cardiovascular signs

Variable *HIE II & III*	B	SE	OD (95% Confidence Interval)	p
**Peripheral** **perfusion**	0.363	0.712	1.438 (0.36 – 5.81)	0.610
**Pulse volume**	1.517	0.673	4.558 (1.22 – 17.05)	**0.024**
**Shock**	-1.489	1.331	0.226 (0.02 – 3.06)	0.263
**Oxygen saturation**	-.010	0.038	0.990 (0.92 – 1.07)	0.780

Reference category: HIE stage I, B* Regression coefficient, OD* Odd ratio

### Relationship between stages of HIE and mortality

Five of the 85 patients with HIE died, giving a case fatality of 5.9%. The case fatality was highest with stage III disease (40.0%) and lowest with stage I (0.0%). Hypoxic ischaemic encephalopathy stage III constituted the highest proportion of 4 (80.0%) of the total mortality. There was a significant difference between stages of HIE and mortality, (p < 0.001), Table 6.

**Table 6:** Relationship between stages of HIE and mortality

Disease stage	Outcome at discharge		*χ* ^2^	*p* value
	Survived n (%)	Died n (%)		
**HIE stage I**	23 (100.0)	0 (0.0)	23.95	**<0.001**
**HIE stage II**	51 (98.1)	1 (1.9)		
**HIE stage III**	6 (60.0)	4 (40.0)		
**Total**	80 (94.1)	5 (5.9)		

HIE: hypoxic ischaemic encephalopathy

Regression analysis of significant cardiovascular signs with mortality

Table 7 shows binary logistic regression analysis of the significant cardiovascular signs with mortality. None of the signs was a significant predictor of mortality.

**Table 7:** Regression analysis of significant cardiovascular signs with mortality

Variable	B	SE	*p*	OD (Confidence Interval)
**Peripheral perfusion**	-1.221	1.588	0.442	0.295 (0.13 – 6.631)
**Pulse volume**	-0.727	1.564	0.642	0.483 (0.02 – 10.36)
**Shock**	0.131	1.610	0.935	1.140 (0.49 – 26.7)
**Oxygen saturation**	0.036	0.042	0.394	1.036 (0.96 – 1.13)

Reference category = Survival, B* Regression coefficient, OD *odd ratio

## Discussion

Oxygen homeostasis is critical for survival and function of all cells in the body including the myocardium. Hypoxia is characterized by inadequate oxygen delivery to the myocardium.^2^

Myocardial hypoxia/ischaemia following perinatal asphyxia may manifest by changes in the heart rate, poor peripheral perfusion, and rise in systemic and central venous pressure. As the myocardium fails, central venous pressure rises further, systemic pressure decreases, and the heart rate drops further.^2^ The mean respiratory rate (66.00 breaths/minute) found in asphyxiated newborn babies with HIE in the present study is similar to 68.00 breaths/minute earlier reported among asphyxiated neonates in the United States.^17^ Also, the mean heart rate found in the current study compares with 145 beats per minute and 146 beats per minute reported in the earlier studies.^17^,^18^

The mean systolic blood pressure (SBP) of 58.47 mmHg herein reported is close to 66.00 mmHg reported by Hall et al.^17^ However, it is much lower than 99.10 mmHg, 76.50 mmHg, and 71.70 mmHg reported in another study, with measurement obtained at less than 15 minutes, 15 to 30 minutes and 30 to 60 minutes of life respectively.^19^ It is this methodological difference from the current study, which obtained blood pressure recordings up to the sixth hour of life, that probably explains the difference in observations. It has been shown that there is a hypertensive response soon after a perinatal asphyxia event: this is followed by progressive reduction in blood pressure.^2^ Regarding diastolic blood pressure and mean arterial blood pressure, the mean values of 28.04 and 38.18 mmHg in the present study are much lower than 42.00 and 49.00 mmHg reported by Hall et al^7^ at less than two hours of life.^2^ It would be pertinent to explore possible methodological reasons for this difference but the earlier report did not indicate the method of blood pressure measurement. This is important since significant differences in diastolic blood pressure have been reported between oscillometric and auscultation methods.^20^

Hypotension was recorded in 52.9% while hypertension was documented in 1.2% of the subjects. Hypotension, being the commonest blood pressure abnormality in the current study may be related to the age at examination (up to six hours). Abnormal blood pressure in asphyxia has been linked to initial non-cerebral vasoconstriction, myocardial ischaemia and eventual heart failure.^2^

Respiratory distress was seen in 64.7% of asphyxiated neonates with HIE. This is within the range of 47.5% to 66.7% reported by other workers.^10^,^21^

Shock was present in 32.9% of the patients which was slightly lower than 45.0% to 48.3% reported by some authors in neonates with moderate to severe asphyxia.^21^,^22^ The lower incidence may be related to more newborn babies with mild to moderate asphyxia that constituted a large number (88.2%) in the current study. In contrast, Rajakumar et al^10^ in India observed a lower incidence of 16.7% despite a similar study population. The higher incidence in the present study may be related to a larger sample size which was nearly thrice the number of new-born babies in the Indian study. Shock may be related to preferential shunting of blood, myocardial ischaemia, vasodilatation and blood loss.^2^ The presence of shock in asphyxiated new-borns may be associated with worse outcome and, as such, is an important clinical cardiovascular finding.^23^

Cardiac failure was present in 5.9% of the patients. This is comparable to 7.5% reported among asphyxiated neonates in India.^21^ It is, however, higher than figures of 1.6% and 2.5% reported by other workers.^24^ On the other hand, it is considerably lower than 36.7% observed by Zhu and Nie.^25^ The incidence reported in the current study may be related to the study population as 11.8% had severe HIE stage. Cardiac failure is most common in the late stage of HIE as the heart is preserved in the early phase of perinatal asphyxia.^2^

Systolic murmurs were present in 9.4% of the patients This is close to 10.0% reported by other workers.^24^ In contrast, it is considerably lower than 20.0% reported by Rajakumaret al.^10^ Systolic murmurs often result from tricuspid insufficiency following an increase in the pulmonary pressure.^23^ It is important to note that systolic murmurs may be a normal finding in the new-born babies, as 21 per 1000 live births have been shown to have murmurs at birth without any significant cardiac abnormalities.^26^

Reduced peripheral perfusion was reported in about half of the patients, which is similar to 43.4% documented in an earlier study.^25^ Reduced peripheral perfusion occurs as a result of preferential shunting of blood so as to preserve the vital organs in the initial phase of asphyxia.^2^ Also, with prolonged asphyxia, heart failure ensues leading to poor circulation.^2^ Low oxygen saturation (SPO2 ≤ 94%) was reported in 48.2% of the patients and may be related to poor peripheral perfusion. This is considerably lower than the incidence of 80.0% reported in the first 15 minute of life in another study.^27^ Since new-borns in the present study were examined at higher postnatal ages of up to six hours, they would be expected to have higher oxygen saturation. The increasing incidence of abnormal cardiovascular signs with HIE severity in this study is consistent with findings by other authors.^10^,^24^ The documentation of pulse volume as the only parameter with an independent relationship with HIE severity in the present study is not out of place, as most of the significant parameters have a relationship with pulse volume.

None of the clinical signs was a significant predictor of mortality. It is difficult to compare our findings on the relationship between cardiovascular signs and stages of HIE because other studies did not subject these parameters to statistical tests.

## Conclusion

The significant cardiovascular signs of clinical interest in HIE were poor peripheral perfusion, hypoxemia, small pulse volume, shock and heart failure. Pulse volume was the only parameter with independent relationship with HIE severity. None of the prarameters had any relationship with mortality. Identification of these signs may be a pointer to myocardial related ischaemic injury in asphyxiated neonates.

A large scale study among term asphyxiated babies with documentation of blood gases to assess the severity of metabolic acidosis is recommended. Electrocardiography and echocardiography might enhance our understanding of the cardiovascular status among the asphyxiated babies.
